# Putting a Kink in HIV-1 Particle Infectivity: Rocaglamide Inhibits HIV-1 Replication by Altering Gag-Genomic RNA Interaction

**DOI:** 10.3390/v16091506

**Published:** 2024-09-23

**Authors:** Paul Rosenfeld, Gatikrushna Singh, Amanda Paz Herrera, Juan Ji, Bradley Seufzer, Xiao Heng, Kathleen Boris-Lawrie, Alan Cochrane

**Affiliations:** 1 Institute of Medical Sciences, University of Toronto, Toronto, ON M5S 1A8, Canada; paul.rosenfeldl@utoronto.ca; 2 Department of Veterinary and Biomedical Sciences, University of Minnesota, Saint Paul, MN 55108, USA; gsingh@umn.edu (G.S.); bseufzer@umn.edu (B.S.); kbl@umn.edu (K.B.-L.); 3 Department of Biochemistry, University of Missouri, Columbia, MO 65211, USA; amp7dp@mail.missouri.edu (A.P.H.); jij@missouri.edu (J.J.); hengx@missouri.edu (X.H.); 4 Department of Molecular Genetics, University of Toronto, Toronto, ON M5S 1A8, Canada

**Keywords:** ribonucleoprotein assembly, eIF4A1, guanosine-adenosine motif, RNA structure

## Abstract

Our examination of RNA helicases for effects on HIV-1 protein production and particle assembly identified Rocaglamide (RocA), a known modulator of eIF4A1 function, as an inhibitor of HIV-1 replication in primary CD4^+^ T cells and three cell systems. HIV-1 attenuation by low-nM RocA doses was associated with reduced viral particle formation without a marked decrease in Gag production. Rather, the co-localization of Gag and HIV-1 genomic RNA (gRNA) assemblies was impaired by RocA treatment in a reversible fashion. Ribonucleoprotein (RNP) immunoprecipitation studies recapitulated the loss of Gag-gRNA assemblies upon RocA treatment. Parallel biophysical studies determined that neither RocA nor eIF4A1 independently affected the ability of Gag to interact with viral RNA, but together, they distorted the structure of the HIV-1 RNP visualized by electron microscopy. Taken together, several lines of evidence indicate that RocA induces stable binding of eIF4A1 onto the viral RNA genome in a manner that interferes with the ordered assembly of Gag along Gag-gRNA assemblies required to generate infectious virions.

## 1. Introduction

As obligate parasites, retroviruses are dependent upon host machineries for expression of viral RNA, synthesis of viral proteins, as well as co-localization of virion genomic RNA (gRNA) and virion protein compositions for infectious particle production [1,2]. Therapies modulating host cell machineries in a manner that interferes with their use by viruses have the potential to serve as antiviral barriers that may be applicable to multiple different viruses reliant on similar host events [3,4]. Host-centric therapeutic strategies thwart the development of resistant viruses, whereas virus-centric therapeutic strategies promote the outgrowth of virions that are drug-resistant. Recent studies have validated this host-centric approach to attenuate virus infection by demonstrating that modulators of the host RNA splicing, translation, lipid metabolism, or cell signaling machineries manifest antiviral barriers [1,5,6,7,8] and provide tools to understand how viruses usurp host mechanisms for their replication.

In the case of HIV-1, studies have uncovered details of host processes following the integration of the provirus into the host genome, including transcriptional trans-activation, balanced viral RNA splicing into multiple RNA isoforms, nucleocytoplasmic transport, and specialized translation unaffected by mTOR [8,9,10]. Many of the processes have been shown to require host RNA helicases, examples of which are the requirement of DDX1 or DDX3 for nuclear RNA export and DHX9/RHA for epigenetic modification of the RNA cap that licenses specialized translation of virion structural proteins [11,12,13,14,15,16,17]. Better understanding of the role played by individual host factors in facilitating each stage of HIV-1 replication offers opportunities for the development of multifaceted strategies to thwart virus growth.

It has been suggested that the extensive RNA secondary structures present within the untranslated region (UTR) of HIV RNAs may render them dependent upon the activity of host helicase eIF4A1 to permit ribosome translocation [14,18,19]. To test this hypothesis, we examined the effect of the eIF4A1 inhibitor, Rocaglamide (RocA), on the replication of HIV-1. While our studies demonstrate that nM-doses of RocA can effectively suppress HIV-1 replication in several experimental systems, the compound did not reduce the expression of proviral RNA nor its translation into viral proteins. Rather, RocA treatment disrupts the assembly of infectious HIV gRNA ribonucleoproteins (RNPs) by altering the subcellular co-location of the gRNA and Gag protein components. Parallel in vitro studies with purified eIF4A1, Gag, and RNA documented that RocA stabilizes the interaction of eIF4A1 with viral RNA non-specifically. While the addition of both eIF4A1 and RocA did not alter the ability of Gag to bind and oligomerize on viral RNA, electron microscopy revealed significantly altered structures were formed. Taken together, by locking eIF4A1 onto the RNA, RocA disrupts the ordered assembly of Gag on gRNA to ultimately inhibit infectious virion formation. Hence, RocA significantly diminishes the transmission of infectious virions from HIV-1 producer cells.

## 2. Materials and Methods

### 2.1. Cells and Compounds

Human primary CD4^+^ cells were isolated from freshly collected blood of normal healthy blood donors obtained from the Memorial Blood Centers by negative selection (EasySep 19052, Stem Cell Technology, Vancouver, Canada) according to the manufacturer’s instructions. The Jurkat derived T cell line JLat 10.6, which contains one latent HIV-1 provirus in which GFP replaces nef and frameshift mutation disrupts the env open reading frame (ORF), was obtained through the NIH AIDS Reagent Program (Division of AIDS, NIAID, NIH, Germantown, MD, USA) [20]. Human lymphocyte line CEM×174 was obtained from ATCC. Low-passage cultures were used in the experiments, and mycoplasma testing was performed periodically. Cell lines were grown in RPMI 1640, 10% FBS, 100 μg/mL Pen-Strep, and 0.5 μg/L gentamycin. The latent provirus in JLat 10.6 was induced by 1.1 µM prostratin (Sigma-Aldrich, St. Louis, MO, USA, Cat. #P0077).

The U2OS cell line HIV-1 FSGagGFP RevGR contains integrated HIV-1 provirus that encodes gag fused in frame with GFP, whereas the rev ORF is disrupted by a frameshift mutation. Rev is provided from a vector expressing a fusion of Rev and the hormone-binding domain of the glucocorticoid receptor [21] (Rev-GR). Supplementation with 25 µM dexamethasone (Dex, Sigma-Aldrich) induces the nuclear import of Rev-GR for Rev/Rev response element (RRE)-mediated nuclear export of US and SS proviral transcripts to the cytoplasm. U2OS cells were maintained in IMDM medium containing 10% FBS, 100 μg/mL Pen-Strep, and 0.5 μg/mL gentamycin. 

Human primary CD4^+^ or CEM×174 cells were seeded into 12-well plates (5 × 10^5^ cells in 1 mL RPMI per well) and spinoculated for 2 h with HIV^NL4−3^ that had been propagated in HEK293 cells, as described previously [22]. Fractions of cultures (10 to 50%) were collected and replenished by fresh culture medium with or without RocA (Rocaglamide, Sigma-Aldrich Cat. # SML0656), at each consecutive time point. Supernatant Gag released into the culture medium was measured by Gag p24 ELISA (XpressBio, Frederick, MD, USA). Viable cells were enumerated by MTT colorimetric assay. Cycloheximide (CHX), puromycin (puro), and actinomycin D (act D), were purchased from Sigma-Aldrich.

### 2.2. Immunoprecipitation of RNP

U2OS or JLat 10.6 cells (1.6 × 10^7^ cells) were treated with 25 µM dexamethasone (U2OS) or 1 µg/mL prostratin (JLat10.6), respectively, for 24 h, medium was removed, the cells were washed three times with cold (4 °C) PBS and then incubated with cold cytoplasmic lysis buffer (10 mM KCl, 0.1 mM EDTA, 10 mM HEPES (pH 7.9), 0.4% NP-40, 1 mM DTT, cOmplete^TM^, EDTA-free protease inhibitors (Roche), SUPERas-in RNAse inhibitor (Ambion)). The cells were gently scraped and incubated in sterile 1.5 mL microtubes on ice for 10 min followed by centrifugation at 18,100× *g* for 10 min at 4 °C, and the nuclear pellet was discarded. GagGFP in the supernatants (cytoplasmic lysates) was quantified by Western blot with anti-GFP antibody (Roche, Cat. #11814460001). For immunocapture of GagGFP, 100 µL of Bio-Rad Sure Beads Protein G (10 µg/µL, 6 µg/mg binding capacity) were washed three times with 500 µL sterile filtered PBS-T (PBS, 0.1% Tween-20) and suspended in 200 µL PBS-T and incubated with the following antibodies for 10 min at room temperature with rotation: GFP antibody 15 µL [0.4 µg/µL] (Roche, Cat. #11814460001); IgG: 1.01 µL [5.9 µ/µL] (Jackson ImmunoResearch, Cat. #711-036-152). Beads were subsequently washed thrice with 500 µL sterile filtered PBS-T, and cytoplasmic lysates were added and incubated overnight at 4 °C with end-over-end rotation. Ten percent of the input cytoplasmic lysate was set aside for downstream protein and RNA analysis. Immune complexes were captured by magnetic separation, washed twice with 200 µL high salt buffer (20 mM HEPES (pH 7.9), 0.4 M NaCl, 1 mM EDTA, 1 mM DTT, Roche protease inhibitor cOmplete) and twice with 200 µL with cytoplasmic lysis buffer. Bead suspensions were divided into two equal volumes. For protein analysis, bead complexes were re-suspended in 50 µL cytoplasmic lysis buffer or RIPA buffer (50 mM Tris-HCl (pH 7.5), 150 mM NaCl, 1% NP-40, 0.5% sodium deoxycholate, 0.1% SDS) and processed for western blotting. For RNA analysis, beads were treated with TRIzol (Invitrogen Cat. 15596026) as per manufacturer instructions, and the RNA samples were stored at −20 °C.

### 2.3. Immunofluorescence Image Analysis

U2OS cells on cover slips (2 × 10^5^) were washed once with PBS, fixed for 10 min in 3.7% paraformaldehyde in 1× PBS at room temperature, washed with PBS, and incubated in 70% EtOH overnight. Coverslips were incubated in wash buffer (10% formamide in 2× SSPE (0.002 M EDTA, 0.298 M NaCl, 0.02 M phosphate buffer (pH 7.4)) for 5 min then inverted on top of hybridization buffer (10% dextran sulfate, 2× SSPE, 10% formamide) supplemented with Stellaris fluorescence in situ hybridization probes (smFISH) conjugated to Quasar 570 (Biosearch Technologies) targeting the *gag* coding region [23] and incubated overnight in the dark at 37 °C within a humidified chamber. Thereafter, coverslips were washed twice with wash buffer for 30 min at 37 °C and then mounted onto slides with Invitrogen^TM^ ProLong^TM^ Gold Antifade mounting buffer with DAPI (Life Technologies). Images were acquired on Leica DMR or Zeiss microscope at 630× magnification using either Openlab imaging software version 2.0.7 or Image J/Fiji v. 2.0.0-rc-68/1.53c.

### 2.4. Concentration of Lentiviral Vector Viruses

As previously described [24], virions produced from JLat10.6 or U2OS cells were isolated from cell free-medium by centrifugation for 2 min at 3000× *g*. After passage through 0.45 μm syringe filters, the clarified media was layered onto 20% sucrose in 50 mM Tris-HCl (pH 7.4), 100 mM NaCl, 0.5 mM EDTA, and centrifuged for 1.5 h at 10,000× *g* at 4 °C. After supernatants were removed, tubes were inverted on tissue paper. The pelleted lentivirus was resuspended overnight at 4 °C in 100 µL PBS.

### 2.5. RNA Isolation, Reverse Transcription (RT), and qPCR Analysis

RNA was isolated in TRIzol and 2 µg aliquots were reverse transcribed to cDNA by M-MLV Reverse Transcriptase (Invitrogen Cat. 28025013), as previously described [25]. Real time PCR was carried out using Taq DNA Polymerase (ABM Life Science Products Cat.#G008) and SYBR (Sigma-Aldrich, Cat.#S9430) with cycling parameters of: 95 °C for 3 min; 40 cycles of 95 °C for 15 s, 55 °C for 20 s, and 68 °C for 30 s. The HIV-1 specific primers were: (genomic sense: 5′-CTGAAGCGCGCACGGCAA-3′, genomic antisense: 5′-GACGCTCTCGCACCCATCTC-3′); SS RNA: (SS sense: 5′-GGCGGCGACTGGAAGAAGC-3′, SS antisense: 5′-CTATGATTACTATGGACCACAC-3′; and MS RNA (MS sense: 5′-GACTCATCAAGCTTC TCTATCAAA-3′, MS antisense: 5′-AGTCTCTCAAGCGGTGGT-3′). Primers specific to GAPDH loading control were: (GAPDH sense: 5′-CATCAATGACCCCTTCATTGAC-3′, GAPDH antisense: 5′-CGCCCCACTTGATTTTGGA-3′).

### 2.6. Protein Analysis

Cell lysates and IP samples were treated for 5 min at 95 °C in 5× dissociation buffer (5% SDS, 50% glycerol. 0.1% bromophenol blue, 250 mM Tris-HCl (pH 6.8)) containing 5% β-mercaptoethanol and analyzed by TGX 10% Stain-Free^TM^ FastCast^TM^ Acrylamide SDS-PAGE (Bio-Rad). Imaging of total protein and GFP signal utilized the ChemiDoc^TM^ MP Imager (Bio-Rad). Proteins were transferred to PVDF membranes (0.45 μm, Perkin-Elmer, Cat. NEF1002) by Trans-Blot Turbo^TM^ blotting system (Bio-Rad). Membranes were incubated in blocking buffer (5% Milk, 0.05% Tween-20 in PBS) for 1 h and overnight at 4 °C after addition of either mouse anti-HIV-1 Gag-p24 hybridoma 183 (1:300 dilution) (NIH AIDS Reagent Program); Lamin A/C (1:5000) (BD Transduction Laboratories, Cat. 612162); α-Tubulin (Sigma-Aldrich, Cat. T9026). Blots were subsequently incubated with HRP-conjugated secondary antibody (1:5000 dilution) (Jackson immunoresearch anti-rabbit Cat.#715-036-150 or anti-mouse Cat.#711-036-152) for 1–2 h and signals visualized with Clarity Western ECL Substrate (Bio-Rad Cat.# 1705060) or Western Lightning ECL Pro (GE Healthcare Cat.# NEL120E001EA).

### 2.7. Recombinant Protein Production and Purification

The eIF4A1 expression plasmid KE-005 was a gift from Gerhard Wagner (Addgene plasmid # 133440; http://n2t.net/addgene:133440, accessed on 5 October 2020). To express recombinant eIF4A1 protein, *E. coli* BL21 (DE3) cells transformed with plasmid KE-005 were grown at 37 °C to an optical density at 600 nm of 0.6–0.8 and induced with 0.5 mM isopropyl-β-D-thiogalactopyranoside (IPTG) for 6 h at 37 °C. Cells were harvested, resuspended in 25 mM HEPES (pH 7.5), 500 mM NaCl, 5 mM imidazole, and 0.5% (*v/v*) NP-40, 1 mM phenylmethylsulfonyl fluoride (PMSF) and 5 mM β-mercaptoethanol, and lysed by sonication. After centrifugation at 13,000 rpm, the supernatant was filtered (0.45 μm) and applied to HisTrap affinity chromatography (GE Healthcare). The protein was eluted by 200 mM imidazole in 25 mM HEPES (pH 7.5), 100 mM NaCl and 5 mM β-mercaptoethanol. The recombinant protein was further purified by size exclusion chromatography (GE Healthcare) and stored in buffer containing 10 mM HEPES (pH 7.5), 10 mM NaCl, 1 mM MgCl_2,_ and 140 mM KCl at −80 °C.

The CA-NC expression plasmid pGEX-CA-NC was built by inserting the HIV-1^NL4−3^ CA-NC (P133 to N432) into parental plasmid pGEX-6-1 (GE Healthcare). To express recombinant CA-NC protein, *E. coli* BL21 Rosetta cells transformed by pGEX-NC-CA plasmid were grown at 37 °C to an optical density at 600 nm of 0.6–0.8 and induced with 0.5 mM IPTG for 20 h at 18 °C. Cells were harvested, resuspended, and lysed by sonication in buffer containing 20 mM Tris-HCl (pH7.5), 50 mM NaCl, 0.1 mM ZnCl_2_, 10 mM β-mercaptoethanol, 1 mM PMSF, and cOmplete^TM^ protease inhibitor (Roche). The supernatant was collected, slowly titrated with 0.11 volume of 2 M (NH_4_)_2_SO_4_ and 10% polyethyleneimine (pH 8) (*v/v* = 10% of lysate) on ice to precipitate nucleic acids. The nucleic acids were removed by centrifugation at 13,000 rpm at 4 °C for 30 min. The supernatant was collected, and the proteins were slowly precipitated by adding 0.35 volume of 2 M (NH_4_)_2_SO_4_. The samples were then centrifuged at 10,000 rpm for 30 min at 4 °C, and the CA-NC containing pellet was resuspended and dissolved in 40 mL of buffer containing 20 mM Tris-HCl (pH 7.5), 100 mM NaCl, 0.1 mM ZnCl_2_ and 10 mM β-mercaptoethanol. The soluble CA-NC protein was further purified by ion exchange chromatography on a SP column with a NaCl gradient from 50 mM to 1 M. The purity of CA-NC protein was confirmed by SDS-PAGE, and the protein was stored in storage buffer (10 mM HEPES (pH 7.5), 10 mM NaCl, 140 mM KCl, 1 mM MgCl_2_, and 10 mM β-mercaptoethanol) in −80 °C.

### 2.8. In Vitro Transcription and RNA Purification

The CES, GA1, GA2, PBS-segment and tRNA^Lys3^ was prepared by T7 in vitro transcription of HIV-1 DNA templates as previously described [26]. The template for tRNA^Lys3^ transcription was synthesized (IDT). GA1 and GA2 mutations were introduced by mutagenesis [27]. The in vitro transcription yields were carried out by mixing DNA template (100 nM), rNTP (12 mM), and T7 polymerase in transcription buffer containing 40 mM Tris-HCl (pH 8.0), 5–10 mM MgCl_2_, 10 mM spermidine, 5 mM dithiothreitol (DTT), and 0.01% (*v/v*) Triton X-100. The mixture was incubated in 37 °C for 3 h and quenched by 25 mM EDTA and 1 M Urea. The RNAs were purified from denaturing polyacrylamide gels, electroeluted by elutrap, and washed with 2 M NaCl to remove residue acrylamide, followed by eight ddH_2_O washes in Amicon centrifugal filters (MilliporeSigma, Burlington, NJ, USA).

### 2.9. Gel Shift Assays

One µM RNA stocks were prepared by boiling the RNA in 10 mM Tris-HCl (pH 7.5) for 3 min and snap cooling on ice for 10 min, then mixed with salts to a final concentration of 140 mM KCl, 10 mM NaCl, and 1 mM MgCl_2_. The mixture was incubated at 37 °C for 1 h. To examine the protein:RNA interactions, eIF4A1 and/or CA-NC were titrated at various ratios into RNA samples to reach final RNA concentration of 0.5 µM in the absence and presence of 50 µM RocA and/or 1 mM AMP-PNP as indicated. Mixtures were incubated at 37 °C for 15 min, loaded onto 1% native agarose gels, and electrophoresed at 120 volts for 45 min on ice. To examine the possible CA-NC:eIF4A1 interactions, the buffer was exchanged by replacing 10 mM Tris-HCl with 10 mM HEPES (pH 7.5), and RNA was refolded in HEPES containing buffer. The protein:RNA mixtures were cross-linked by 0.12% glutaraldehyde at 37 °C for 5 min, and the reactions were quenched by adding 100 mM Tris-HCl. The samples were visualized by 4–20% gradient SDS-PAGE. 

### 2.10. In Vitro Assembly of RNPs and Electron Microscopy

Assembly reactions were prepared using 20 µM CA-NC, 1 µM CES RNA, 1 mM AMP-PNP, 40 µM eIF4A1, and 50 µM RocA (unless otherwise indicated). Samples were dialyzed against assembly buffer (50 mM Tris-HCl (pH 8.0), 100 mM NaCl) for 2 h at 4 °C in Slide-A-Lyzer (Themofisher #69570). When eIF4A and RocA were used in assembly reactions, the samples were preincubated without CA-NC for 30 min at room temperature. After the preincubation, CA-NC was added to the specified concentration and the samples were dialyzed as described. 

Following dialysis, assembly reactions (5 µL) were deposited on carbon-coated 200 mesh copper grids (Electron Microscopy Sciences) that were freshly glow discharged for 45 s, 0.39 mBarr at 15 microamps by a Pelco Easiglow Glow Discharge Cleaning System (Ted Pella). The specimens were incubated on the grid for 2 min, then wicked by filter paper (Whatman P1). Immediately, samples were stained with 5 µL NanoW (Nanoprobes) for another 2 min and wicked dry. The specimens were visualized on a JEOL JEM-1400 Transmission Electron Microscope operated at 120 kV. Micrographs were acquired manually using different magnifications on a Gatan Ultrascan 1000 (Gatan, Inc., Pleasanton, CA, USA) CCD camera. 

## 3. Results

### 3.1. Rocaglamide Suppresses HIV-1 Replication

**Figure 1:**
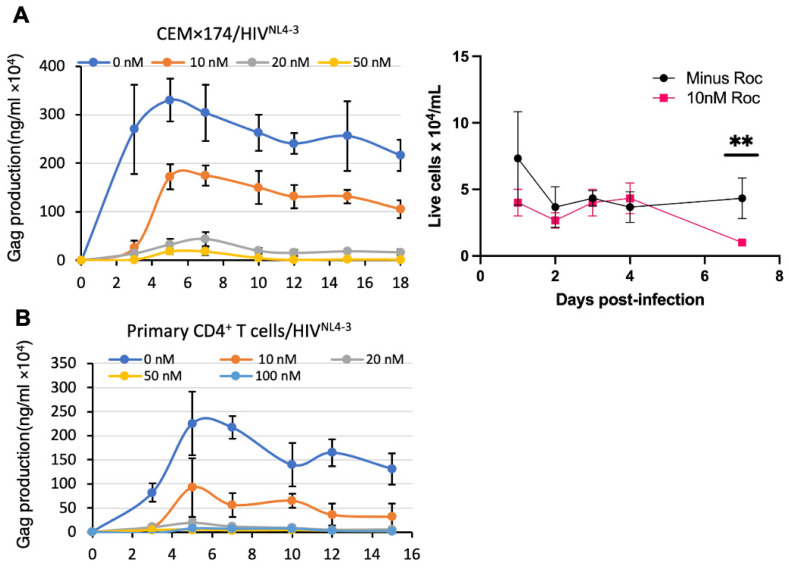
RocA inhibits HIV -1 replication. To assess the effect of Roc on HIV proliferation, (**A**) CEM×174 lymphocytes or (**B**) primary CD4^+^ T lymphocytes were infected with HIV^NL4−3^ for 6 h (MOI = 1), washed and then continuously cultured with or without RocA (0, 10, 20, 50 nM). Cell-free supernatants were collected at regular intervals and virus proliferation measured by Gag ELISA (filled shapes). To assess effects of RocA on cell viability ((**A**), **right**), CEM×174 lymphocytes were cultured in medium with RocA (red squares) or without (black circles) (2 × 10^6^ cells/mL in 6-well plate). Viable cells were enumerated at day 1, 2, 3, 4, 7 using trypan blue ((**A**), **right**). At 3 day intervals, 50% of each culture was collected and replaced with fresh uninfected cells. Data points are the mean and standard deviation of three replicate wells. Students’ *t* test documented no significant difference (*p* = 0.15–0.2, day 1–4, *p* = 0.06, day 7) minus and plus Roc at each day. 95% confidence, significance cutoff *p* < 0.05.

To examine whether rocaglamide (RocA) could affect HIV-1 replication, a dose-response analysis was performed in primary CD4^+^ T cells and CEM×174 lymphocytes. The cells were spinoculated with HIV^NL4−3^ and cultured in medium with vehicle (DMSO), 10, 20, or 50 nM RocA. At 2 to 3-day intervals, 50% of the culture was collected and cell-free medium was subjected to Gag ELISA. The remaining cultures were replenished with uninfected cells in medium supplemented with DMSO and RocA. The spread of virus infection between the mock treatment and 10 nM RocA treatment was attenuated, both in the magnitude and rate of virus growth in CEM×174 (Figure 1A, left) and primary CD4^+^ T cells (Figure 1B) over several passages. In these infections, virus production peaked after day 2, and by day 5, cell viability within this same time frame remained similar between DMSO and 10 nM RocA, diminishing after day 7 (50%). (Figure 1A, right). The spreading infections in 0 nM RocA peaked at day 4, and was reduced 50% by 10 nm RocA treatment, 90% by 20 nM, and completely suppressed by 50 nM RocA in both primary- and cultured CD4^+^ T cells. We concluded that within 4 days, RocA significantly reduces HIV-1 replication.

**Figure 2:**
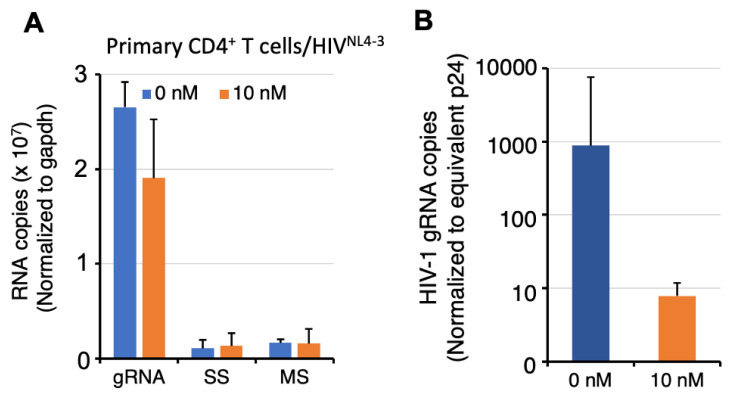
RocA affects HIV-1 gRNA encapsidation. (**A**) RocA does not affect HIV-1 intracellular accumulation. Total RNA was extracted from infected primary CD4^+^ T lymphocytes incubated +/− 10 nM RocA and levels of HIV-1 gRNA, SS, and MS RNA relative to GAPDH RNA was assayed by RTqPCR. (**B**) Virions from RocA treated cells have reduced HIV-1 gRNA content. Virion-associated RNA was extracted from equivalent p24 units of cell-free virions and subjected to RT-qPCR. Roc treatment significantly reduced HIV virion RNA copies, indicating that HIV-1 gRNA packaging was deficient.

To gain insight into the basis for the observed HIV-1 replication defect, subsequent analyses focused on changes in HIV-1 RNA expression in cells and accumulation in virions. Total cellular RNA from the infected primary CD4^+^ T cells treated with DMSO or 10 nM RocA was collected and RT-qPCR performed with primers specific to each of the three classes of HIV-1 RNA isoforms: fully spliced mRNAs encoding Tat, Rev, Nef (MS), incompletely spliced (SS) mRNAs encoding Vpu and Env virion proteins, and the full-length viral gRNA, which are either translated to structural and accessory protein components of virions, or assembled into progeny virions as genomic RNA (gRNA). GAPDH RNA was used as a loading control. As shown in Figure 2A, RT-qPCR of primary CD4^+^ T cells treated with vehicle or 10 nM RocA revealed no alteration in intracellular abundance of the HIV-1 genomic, SS, or MS RNAs, indicating that RocA did not affect proviral RNA processing or steady-state abundance. To assess whether gRNA content of the virions was affected by RocA, the copies of gRNA per equivalent Gag (p24) was examined in virions propagated in primary cells treated in the presence of DMSO or 10 nM RocA. As shown in Figure 2B, the RT-qPCR analysis revealed virion-associated gRNA copies were diminished by a factor of 100 (*p* = 0.001). Given the lack of any change in intracellular HIV-1 gRNA abundance, the reduced gRNA content of viral particles is likely due to a packaging defect. 

### 3.2. RocA Inhibits HIV-1 Virion Formation without Marked Changes in Gag Production

**Figure 3:**
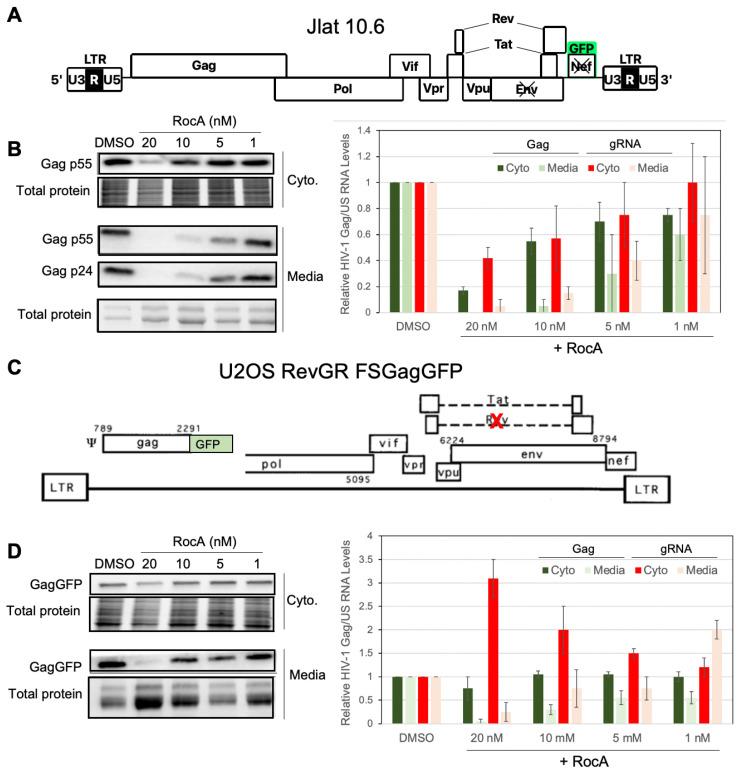
At low doses, RocA has limited effect on HIV-1 Gag expression but blocks Gag and viral RNA release from JLat and U2OS RevGR FSGagGFP cells. To examine the effect of RocA on HIV-1 protein expression and virus assembly, JLat 10.6 (**A**,**B**) and U2OS RevGR FSGagGFP (**C**,**D**) cell lines were treated with vehicle (DMSO) or RocA. Provirus structures for each cell line are provided (**A**,**C**). HIV-1 provirus expression was induced by either addition of prostratin (**B**) or dexamethasone (**D**) in the presence of increasing doses of RocA (1–20 nM) and, after 24 h, media and cytoplasmic lysates (Cyto.) harvested for analysis. Representative western blots of cell lysates and collected virus particles are shown on the left (**B**,**D**) along with total protein loads (Total protein as determined using Stain-free gels). (**B**,**D**) Total RNA was extracted from cytoplasmic lysates (Cyto.) or media (Media) and abundance of HIV-1 gRNA determined by RT-qPCR. Shown on the right are graphs summarizing data from *n* > 3 independent assays reflecting the levels of HIV-1 Gag protein or gRNA in each fraction, values expressed relative to those seen upon addition of DMSO.

To further explore the basis for reduced HIV-1 replication in the presence of RocA, we examined the effect of the compound in two additional experimental systems. The first system was JLat10.6, a Jurkat CD4^+^ T cell line containing a single HIV-1 provirus, wherein the nef open reading frame has been replaced with GFP and the Env open reading frame has been disrupted (Figure 3A) [20]. Expression of the provirus in JLat10.6 was low until the addition of a latency reversal agent such as prostratin. Twenty-four hours following prostratin and RocA treatment, Gag production was evaluated by western blot. As shown in Figure 3B, treatment with 20 nM RocA severely reduced Gag p55 and subtly reduced total cell protein. By comparison, RocA treatment at 10, 5, or 1 nm had little effect on total cell protein. Comparison of cell-associated Gag and accumulated Gag p55/p24 in cell-free medium revealed discordance at the 10 and 5 nM doses relative to or 1 nM RocA, indicative of a block in virus release. Parallel analysis of the HIV-1 RNA in the cytoplasm indicated concordance between Gag protein levels and HIV-1 gRNA abundance, at doses of RocA below 20 nM excluding the possibility RocA diminished steady state gRNA translation (Figure 3B). Parallel analysis of extracellular HIV-1 RNA abundance identified a significant decline in virion-associated gRNA upon treatment with 10 nM RocA (Figure 3B), recapitulating the reduction in gRNA content of virions propagated in the HIV-1 NL4-3 infected primary CD4^+^ T cells (Figure 2B). Taken together, the results suggested the replication defect induced by RocA treatment (10 nM) was attributable to an assembly defect.

To further investigate the basis for the deficient HIV-1 replication caused by RocA, we examined the effect of RocA addition in the context of the U2OS FSGagGFP RevGR cell line, which contains an HIV-1 provirus expressing a GagGFP fusion protein and a frameshift mutation that prevents expression of the rev open reading frame (Figure 3C, Figure S1). Since transport to the cytoplasm of HIV-1 genomic and SS RNA is dependent upon trans-activation by Rev, production of their protein products is dependent upon expression of Rev in trans [28]. In this U2OS system, a second vector constitutively expresses Rev fused to the hormone-binding domain of the glucocorticoid receptor (RevGR), rendering Rev function dependent upon dexamethasone addition to the media [21]. Dose-response analysis with RocA revealed similar trends as in the JLat.10 cells. Treatment with 20 nM RocA slightly reduced Gag and steady-state protein in cells, consistent with diminished cell viability (Figure 3D). Treatment with RocA at 10 nM or 5 nM had no effect on Gag-GFP intracellular abundance but markedly reduced the accumulation of GagGFP in the media. Parallel RNA analysis determined that both 20 nM and 10 nM RocA increased cytoplasmic HIV-1 gRNA levels while 20 nM RocA reduced gRNA accumulation in the medium (Figure 3D).

**Figure 4:**
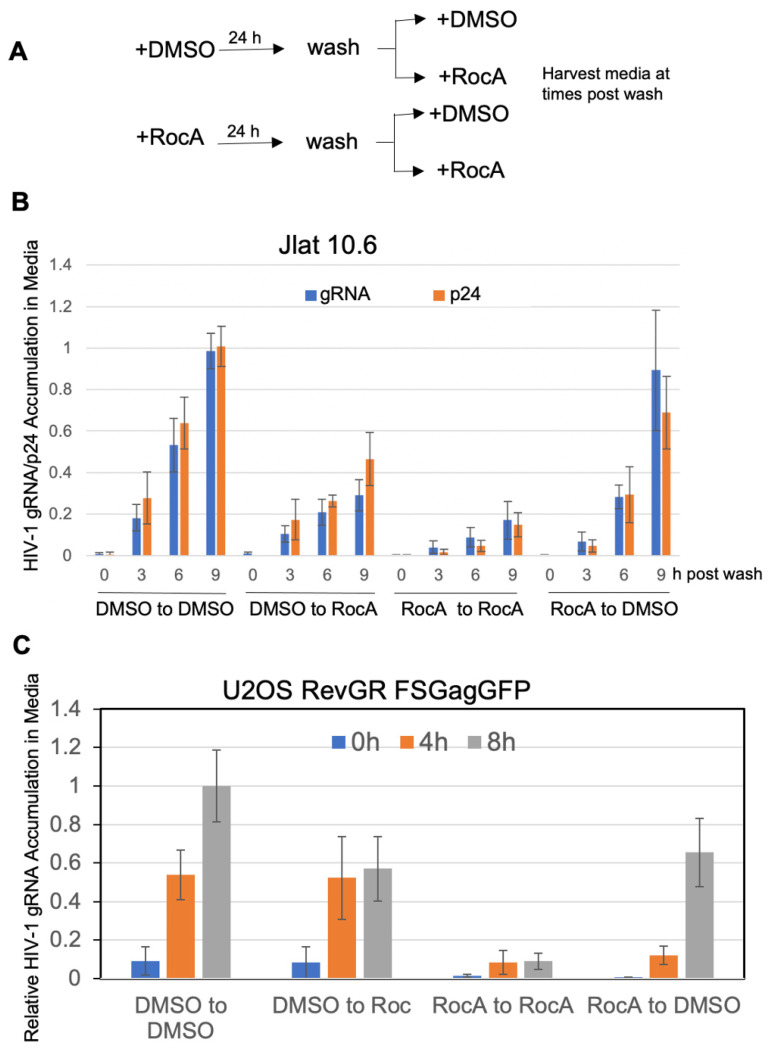
The effect of RocA on HIV-1 virus formation is reversible. To examine the reversibility of RocA’s effect on HIV-1 virus formation, as detailed in (**A**), cells were treated either with DMSO or RocA for 24 h, washed, then incubation continued under the same condition (DMSO to DMSO, RocA to RocA) or switched to the other condition (DMSO to RocA, RocA to DMSO). Aliquots of media were harvested at 0, 3, 6, and 9 h after wash ((**B**), JLat 10.6) or 0, 4, and 8 h post wash ((**C**), U2OS RevGR FSGagGFP) and levels of HIV-1 Gag and viral gRNA measured by p24 ELISA and RT-qPCR, respectively. Graphs shown summarize *n* > 3 independent assays.

In light of the effects of RocA on HIV-1 virion release from cells, we explored the reversibility of the apparent block in both the JLat10.6 and U2OS systems. As outlined in Figure 4A, cells were treated with either vehicle (DMSO) or RocA for 24 h, at which time cells were washed and cultured in fresh media containing either DMSO or RocA. Sampling of media of JLat10.6 over the next 9 h revealed a parallel increase in extracelluar HIV-1 Gag and gRNA in the presence of DMSO, while cells maintained in RocA showed limited accumulation of either (Figure 4B, DMSO to DMSO or RocA to RocA). The addition of RocA to cells previously exposed to DMSO (DMSO to RocA) resulted in a similar extent of cell-free Gag and gRNA accumulation as seen in untreated cells (DMSO to DMSO) for the first 3 h after the media change. However, accumulation of Gag and gRNA was reduced at 6 and 9 h, suggesting that RocA affects de novo assembly. Conversely, cells pretreated with RocA and switched to media containing only DMSO (RocA to DMSO) showed reduced accumulation of both Gag and gRNA in the media relative to control (DMSO to DMSO) at 3 h post-wash, and significant accumulation at later times (6 and 9 h), demonstrating that the block to de novo assembly was reversible. Analysis of the accumulation of HIV-1 gRNA in media in the U2OS HIV FSGagGFP RevGR system (Figure 4C) revealed a similar pattern. Switching the treatment of the cells resulted in a delay in the pattern of gRNA release, mimicking the prior condition for the first 4 h and then switching to the pattern of the new condition. In both the JLAT and U2OS system, the restoration of HIV-1 assembly and release following RocA treatment was dependent on both de novo protein and RNA synthesis, as treatment of cells upon removal of RocA with cycloheximide/puromycin to inhibit protein translation or actinomycin D/5,6-dichloro-1-β-D-ribofuranosyl benzimidazole (DRB) to inhibit transcription resulted in no accumulation of Gag or gRNA in the media (Figure S2). Taken together, these observations indicate that RocA inhibition of virus assembly and release is reversible and experiences a temporal lag of approximately 3–4 h in both systems.

### 3.3. RocA Treatment Affects HIV-1 Gag Protein and gRNA Subcellular Interaction

**Figure 5:**
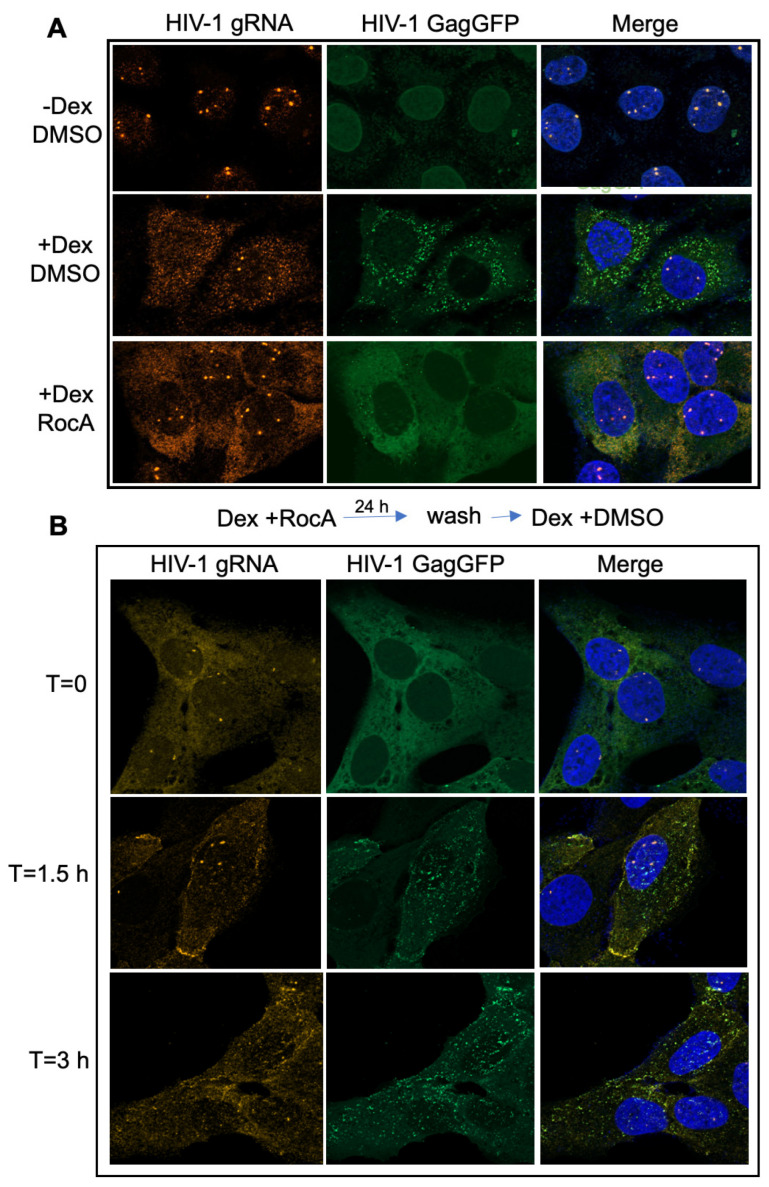
RocA alters cytoplasmic distribution of HIV-1 Gag and genomic RNA in a reversible fashion. (**A**) To examine the effect of RocA treatment on HIV-1 gRNA and GagGFP subcellular distribution, virus expression was induced in U2OS RevGR FSGagGFP cells treated with DMSO or RocA and cells fixed for in situ hybridization/microscopy 24 h after dexamethasone addition. Shown are representative images of results obtained from cells incubated with DMSO (−/+ dexamethasone) (−Dex, DMSO), +Dex, DMSO) or RocA and dexamethasone (+Dex, RocA). Magnification 630×. (**B**) U2OS RevGR FSGagGFP cells were treated for 24 h with dexamethasone and RocA, washed, and incubated with media containing dexamethasone and DMSO. Cells were fixed at 0, 1.5, or 3 h post wash for subsequent detection of HIV-1 gRNA by in situ hybridization. Shown are representative images from *n* > 3 independent assays. Magnification 630×.

To gain greater insight into how RocA might be affecting HIV-1 assembly and release, we examined the effect of RocA on the subcellular distribution of the Gag protein and the proviral gRNA using in situ hybridization with fluorescence probes in the U2OS system. Consistent with a requirement for Rev function, limited cytoplasmic GagGFP was observed in the absence of dexamethasone and the gRNA signal was limited to the nucleus (top panels) (Figure 5A). Previously, we have shown the more intense nuclear foci of gRNA signal are transcription sites [23]. The addition of dexamethasone for 24 h (+Dex, DMSO) resulted in significant accumulation of GagGFP and gRNA in the cytoplasm with colocalization of GagGFP and gRNA in discrete foci that may reflect sites of virion assembly (middle, far right panel). Parallel analysis of cells treated with RocA (for 24 h) (Figure 5A, +Dex RocA) revealed significant accumulation of both GagGFP and gRNA in the cytoplasm (right panels), yet with an altered pattern of distribution relative to control (+Dex DMSO). Rather than localized to foci within the cytoplasm, signals for both were distributed uniformly throughout the cytoplasm, consistent with a dearth of Gag-gRNA interaction that may be necessary for proper virion assembly. To further test the effect of RocA on the subcellular co-localization of GagGFP and gRNA, the reversibility of the RocA treatment was also examined. Cells treated with RocA for 24 h in the presence of dexamethasone were washed to remove the compound, and incubation continued until the samples were fixed at 0, 1.5, or 3 h post-wash (Figure 5B). Examination of the images revealed that 1.5–3 h after RocA removal, co-localization of GagGFP and gRNA in cytoplasmic foci was restored. These results recapitulate the reversibility of RocA inhibition of de novo virus production (Figure 4). The delay in response suggests RocA affects RNA-protein complex assembly near an initial stage, since those complexes already in the process of virion formation were able to complete the assembly and release process (Figure 4B,C).

**Figure 6:**
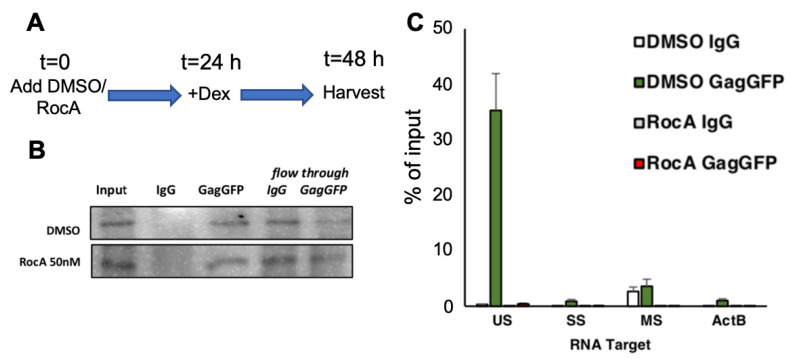
RocA inhibits formation of stable HIV-1 Gag-genomic RNA complexes. (**A**) U2OS RevGR FSGagGFP cells were treated with dexamethasone and either DMSO or RocA. Cells were harvested and cytoplasmic extracts prepared as detailed in Section 2. Shown in (**B**) are representative SDS-PAGE gels of cell extracts before (input) or after immunoprecipitation with control (IgG) or anti-GFP (GagGFP) antibodies as well as flow through following capture of immunocomplexes with protein G magnetic beads. (**C**) RNA extracted from immunoprecipitates recoverd from cells treated for 24 h +/− RocA were used to measure abundance of HIV-1 gRNA (US), singly spliced (SS), and multiply spliced (MS) as well as actin (ActB) mRNA in these samples by RT-qPCR. Shown is a representative result from *n* > 3 assays.

As mentioned above, RocA diminished colocalization of GagGFP and gRNA, suggesting that the compound was affecting the interaction between the RNA and protein components of virions. As a direct test of this hypothesis, RNP immunoprecipitation assays (RIP) were performed using antibody to the GFP portion of GagGFP or IgG control, followed by RT-qPCR (Figure 6A). Western blot validated effective IP of Gag-GFP by GFP, but not IgG antiserum, with some residual Gag-GFP present in the flow through the IP reactions (Figure 6B). Anti-GFP RNP IP on cytoplasmic extracts from the DMSO-treated U2OS HIV FSGagGFP RevGR cells co-precipitated HIV-1 gRNA (US), whereas HIV-1 SS, MS, and actin RNAs were poorly enriched (Figure 6C). As expected, RIP with non-immune IgG poorly enriched HIV-1 gRNA, as well as SS, MS, and actin RNA. Parallel anti-GFP RNP IP on cytoplasmic extracts from RocA-treated cells (Figure 6C) resulted in levels of all RNAs being comparable to control the antibodies. These results confirm that RocA treatment impaired HIV-1 Gag and gRNA interaction in cells. 

### 3.4. RocA and eIF4A1 Alter Gag-Viral RNA Interaction In-Solution

**Figure 7:**
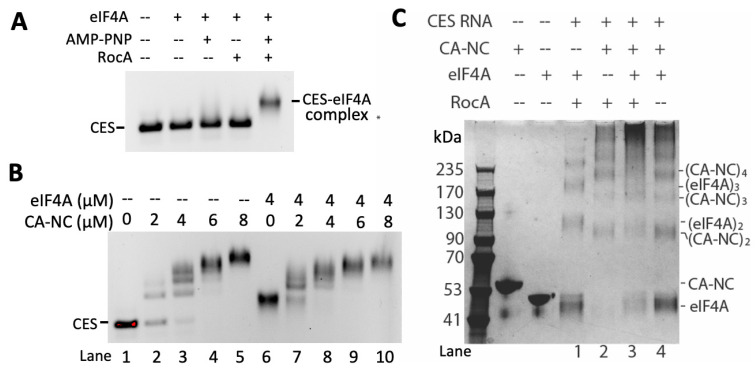
RocA has limited effect on formation of Gag-genomic RNA complexes in vitro. (**A**) eIF4A1 forms a complex with CES RNA only in the presence of both AMP-PNP and RocA. (**B**) 2–8 µM CA-NC cooperatively bound to 0.5 µM CES RNA in the absence (lanes 1–5) and presence (lanes 6–10) of 4 µM eIF4A1. (**C**) CES, CA-NC, eIF4A and RocA were mixed at indicated conditions, cross-linked with glutaraldehyde, and subjected to 4–20% SDS-PAGE. Lane 1, in the presence of RocA, cross-linked eIF4A oligomers were observed. Lane 2, oligomerization of CA-NC was promoted upon RNA binding. Lane 3, higher ordered complexes were formed when both eIF4A and CA-NC were present. Lane 4, in the absence of RocA, the crosslinked protein complexes exhibited similar migration pattern as that of lane 2 with free eIF4A band, suggesting eIF4A did not bind to the complex.

To explore in greater detail the basis for RocA impairment of the interaction between Gag and gRNA, we turned to in vitro binding assays. In recent studies, RocA caused eIF4A1 to clamp onto RNA purine-rich motifs within single-stranded regions [29,30]. Recently, purine-rich motifs were shown to be highly conserved in the 5′ UTR of HIV-1, HTLV-1, and spleen necrosis virus [27]. Mutation of either of two guanine-adenosine motifs flanking the 5′ splice site severely reduces HIV-1 fitness. Since the guanine-adenosine motifs resemble binding motifs preferred by eIF4A1 [27,30], we postulated the motifs may be important for RocA impairing Gag-RNA interaction. To test this hypothesis, we performed electrophoretic mobility shift assays (EMSAs) to evaluate whether eIF4A1 interacts with the HIV-1 5′ UTR. As shown in Figure 7A, formation of a stable complex between recombinant eIF4A1 and RNA containing the core encapsidation signal (CES RNA) was dependent upon RocA and the ATP analogue (AMP-PNP) [31,32]. Next, CES RNAs containing mutations in the conserved guanosine-adenosine motifs were evaluated. The EMSA results were unchanged upon mutation of GA1 motif (GAGGGAGA to GcGGuuA) or GA2 motif (AGGAGAG to AccuGAG) [27], indicating affinity to eIF4AI was similar to wild-type CES RNA (Figure S3A). Consequently, eIF4A1 can bind to regions other than these GA motifs in the 5′UTR. By comparison, eIF4A1 also binds to two regions of SARS-CoV-2 RNA containing guanosine-adenosine motifs (SARS-CoV2-RNA1: nt 29,534–29,658, 126 nt; SARS-CoV2-RNA2: nt 29,621–29,675 and nt 29,831-nt 29,874, connected by a GAGA tetraloop, 116 nt) (Figure S3B), but not tRNA^Lys3^ (Figure S3A), a highly structured RNA that lacks GA-rich sequences in its single-stranded loop. These data suggest that RocA enables eIF4A1 to non-specifically bind to RNAs with purine-containing flexible loops.

To determine whether eIF4A1 binding to CES RNA would disrupt the direct interaction between CES RNA and Gag, recombinant Gag domains were expressed and purified. Gag is composed of matrix, capsid, and nucleocapsid domains (MA-CA-NC). When increasing concentrations of the CA-NC portion of Gag were incubated with CES RNA, cooperative binding activity was observed, indicated by multiple bands detected at CA-NC:CES ratios of 1:4 and 1:8 (Figure 7B, lanes 2, 3). The binding cooperativity may be due to the CA domain forming dimers or higher-ordered oligomers upon binding to RNA. We then pre-incubated CES RNA with eIF4A1 plus RocA prior to mixing with CA-NC at various concentrations. Figure 7B lane 6 shows a band higher than free CES in lane 1, demonstrating that eIF4A1 binds to CES RNA. Addition of CA-NC further shifted the complex, consistent with CA-NC interacting with the eIF4A1-CES RNA complex. eIF4A1 did not appear to compete for binding sites with CA-NC, as the cooperative binding pattern of CA-NC was also detected when RNA was pre-bound with eIF4A1. Interestingly, binding of eIF4A1 to CES appeared to increase its affinity for CA-NC. The eIF4A1-CES RNA band produced by 2 µM CA-NC is much less intense (Figure 7B, lane 7) than the free CES RNA band not preincubated with eIF4A1 (Figure 7B, lane 2). A similar trend is also observed when comparing lanes 3 and 8 in Figure 7B, with all of the RNA bound to protein in the presence of eIF4A1 and CA-NC. These data suggest that eIF4A1 binding to CES RNA increases the affinity of CA-NC to CES RNA. 

Replacement of the CES RNA with non-HIV RNA was examined to determine whether the effect is specific to the HIV-1 packaging signal. RNA fragments from SARS-CoV-2 3′UTR (SARS-CoV-RNA1 and SARS-CoV-RNA2) were mixed with eIF4A1 and complex bands were also detected in the EMSA, recapitulating results seen with CES RNA (Figure S3). The CA-NC titration with SARS-CoV-2 RNAs without or with eIF4A1-prebound reiterated that eIF4A1 binding to RNA enhances the affinity of CA-NC even though SARS-CoV-2 RNAs are not the cognate targets of CA-NC. Taken together, the results support the possibility that RocA promotes clamping of eIF4A1 onto RNAs in a nonspecific manner and that eIF4A1 promotes CA-NC binding to RNA in general. 

To test if the CA-NC affinity increase is mediated by protein:protein interactions, the RNA:eIF4A1:CA-NC mixture was cross-linked using glutaraldehyde and samples resolved by SDS-PAGE. Upon RNA binding, both CA-NC and eIF4A1 could form higher-order oligomers (Figure 7C, lane 1 versus 2). In the presence of RocA, when both eIF4A1 and CA-NC were present, the complex was positioned higher than eIF4A1-only or CA-NC-only reactions (Figure 7C, lanes 1–3). However, when mixing eIF4A1, CA-NC, and CES RNA in the absence of RocA, the cross-linked protein pattern was similar to the CA-NC:CES mixture (Figure 7C, lane 4), indicating that, in the absence of RocA, CA-NC cannot be cross-linked with eIF4A1. Thus, the eIF4A1:CA-NC interaction is RNA- and RocA-dependent. 

**Figure 8:**
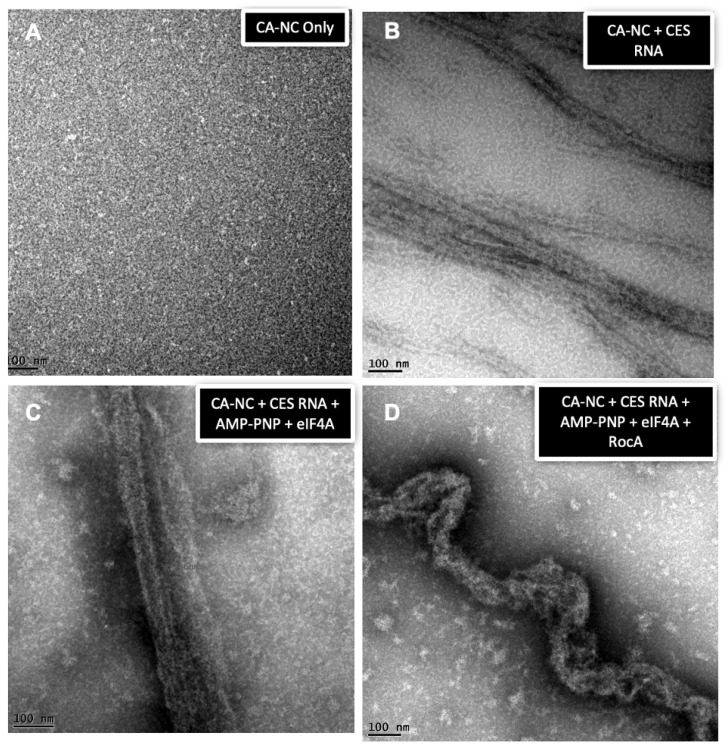
RocA alters the lattice structure of the CA-NC - RNA complexes assembled in vitro. (**A**) Soluble CA-NC protein only. In the absence of RNA, no ordered structures were observed. (**B**) CA-NC assembled into a lattice structure in the presence of RNA. Several tube-like assemblies were observed with varying lengths at a constant 30 nm diameter. (**C**) Addition of eIF4A1 and AMP-PNP and did not alter the tube-like lattice structure of the CA-NC:RNA complexes. (**D**) Addition of eIF4A1 and RocA results in the formation of kinked Gag-CES RNA assemblies.

**Figure 9:**
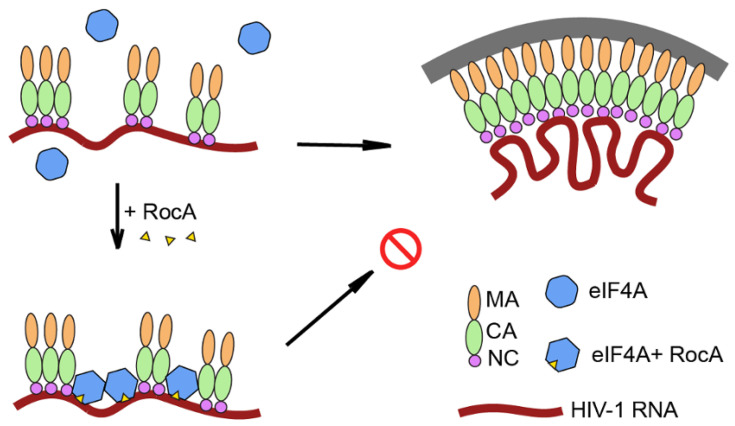
A model of RocA’s effect on HIV-1 particle assembly. In the absence of RocA, HIV-1 Gag oligomerizes along the gRNA, resulting in displacement of any proteins bound to generate the final assembly on the plasma membrane, In the presence of RocA, eIF4A1 becomes bound to the HIV-1 gRNA, blocking the oligomerization of Gag on the gRNA, preventing plasma membrane association and release of the viral particle.

To gain greater insight into the nature of the RNP complexes formed upon incubation of CES RNA with CA-NC and/or eIF4A1, assemblies were analyzed by electron microscopy. As shown in Figure 8A, CA-NC alone was detected in small aggregates. However, as documented previously [33], incubation of CA-NC with CES RNA resulted in the formation of tube-like structures consistent with oligomerization of CA-NC along the RNA (Figure 8B). Addition of eIF4A1 and AMP-PNP to the CA-NC and CES RNA assembly reaction (Figure 8C) yielded tube-like structures similar to those detected in Figure 8B, indicating eIF4A1 was not disruptive to the HIV RNP assemblies. In contrast, addition of RocA to the reaction altered the RNP structure formed (Figure 8D). Rather than the linear tube-like structures formed by CA-NC and CES RNA, addition of both RocA and eIF4A1 generated distorted tubes that appeared helical in nature. Additional representative electron micrographs of CA-NC assembly under these conditions are shown in Figure S4. On average, the CA-NC tube length between kinks (defined as the angles < 150° within the tube structure) in the absence of RocA was 2.34 ± 1.45 µm. When RocA was present, the average length significantly shortened to 0.22 ± 0.06 µm (*p* < 0.0001). Taken together, the results of cell-based assays and in-solution assays with RocA and eIF4A1 suggest the RocA treatment of HIV producer cells attenuated HIV replication by disrupting the ordered assembly of the HIV RNP required for HIV replication (Figure 9). 

## 4. Discussion

Previously, RocA therapeutic activity has been attributed to clamping eIF4A1 on mRNA templates and blocking ribosome activity, thus halting polypeptide synthesis from eIF4F-dependent mRNAs. At the concentrations used to inhibit HIV-1 replication (10–20 nM), our data do not support RocA-inhibiting HIV-1 polypeptide synthesis since Gag protein levels were commensurate with gRNA levels. Instead, RocA antiviral activity was attributable to defective viral particle assembly and release. Moreover, inhibition of eIF4F activity does not affect expression of HIV-1 incompletely spliced templates since they undergo specialized translation licensed by a unique tri-methylated-cap structure and nuclear cap-binding proteins [22]. However, eIF4F activity is necessary for translation of the HIV-1 MS mRNAs [22], thus RocA could downregulate HIV MS mRNA translation. However, downregulation of Tat and Rev reduces gRNA levels [22]. This is not observed over the time frame of the replication assays; we exclude the possibility for it contributing to the significant attenuation of HIV production observed in primary CD4^+^ T cells (Figure 1) [34]. Results in the U2OS system ruled out loss of Rev as a confounder in RocA attenuation of HIV-1. The viral gRNA abundance was not affected in the primary and cultured cells lines used in our study. Additional experimentation would be required to assess whether off-target effects of the RocA treatment compromise host-dependency factors or favor antiviral factors and contribute to the ablation of HIV-1 replication in our experiments.

In situ analysis of HIV-1 infected cells determined that the co-localization HIV-1 Gag and gRNA was significantly affected by RocA. Cell-based biochemical experiments determined RocA treatment blocked the Gag interaction with gRNA in cells. In vitro biophysical experiments provided an explanation for this effect; RocA stabilization of eIF4A1 binding to RNA, albeit nonspecifically. RocA treatment appears to block the HIV Gag—RNA interaction at an initial stage of viral RNP assembly, since wash-out experiments revealed a 3–4 h delay between its addition and disruption of the full assembly process. The reversibility of the RocA block to HIV-1 particle assembly may become a useful tool in studies exploring this portion of the virus life cycle. The ability to rapidly restore HIV-1 virion release upon compound removal indicates that RocA does not irreversibility alter the cellular environment to impair virion formation. However, the failure to restore virion release upon RocA removal in the presence of inhibitors of RNA or protein synthesis (despite the presence of significant quantities of both Gag and viral gRNA in the cytoplasm) unveils a requirement for a highly labile factor and de novo RNA synthesis consistent with prior studies [35]. 

Our in vitro studies investigating the impact of RocA/eIF4A1 on Gag RNP assembly provide some insights into the process. As detailed in Figure 7, eIF4A1 forms a stable complex with RNA only in the presence of RocA and AMP-PNP, oligomerizing on the RNA as indicated by the ladder formed upon cross-linking. Furthermore, in vitro, the interaction of eIF4A1 with RNA does not prevent the association of Gag and its multimerization along the RNA. Rather, the addition of eIF4A and RocA alters the nature of the complex formed between Gag and RNA. EM analysis determined that the linear Gag-RNA assemblies (−eIF4A) are distorted (kinked, +eIF4A) tubes. The formation of the distorted tubes is RocA-dependent as the addition of eIF4A alone generated structures similar to those seen with Gag and RNA, suggesting that the oligomerization of Gag along the RNA can displace any eIF4A bound. Distortion in the RNP in the presence of RocA is expected given that binding of eIF4A in the presence of RocA is known to induce a bend in the associated RNA at the site of binding [30]. Consequently, the preference of eIF4A to bind polypurine sequences [29], coupled with its capacity to bind to multiple points on the RNA, is consistent with the distorted structures observed. In translating these findings to the loss of Gag-gRNA interaction in cells upon RocA addition, it should be noted that in vitro studies detailed here represent a significant simplification of the interactions occurring in the cell given the more limited number of components added (RNA, CA-NC, eIF4A1) in the former. 

The in vitro Gag-RNA and eIF4A-RNA assemblies were observed to form on multiple RNA templates lacking known packaging signals, the exception being highly structured tRNA, indicating a requirement for access to single-stranded regions (Figure S3). As a result, in the cell one might anticipate that multiple, competing interactions are occurring that affect the equilibrium of the interactions under study. The key point that emerges from the in vitro studies is that locking eIF4A1 onto the RNA with RocA addition does not impair the ability of Gag to bind onto the RNA (critical for the initiation of the packaging process) but blocks the formation of an ordered Gag multimer on the RNA. In vitro, the conditions selected strongly favor Gag-RNA assembly while the nonspecific recruitment of Gag to other RNAs was enhanced. In the cell, disruption of the ordered oligomerization of Gag on the HIV RNA likely inhibits the successful assembly of virions and increases access of the viral RNA to other competing processes. The loss of Gag-US RNA co-localization in cells in the presence of RocA can be attributed to either distortion of the RNA structure near the packaging signal that compromises an initial contact with Gag or its ability to oligomerize along the gRNA to form a stable complex required for infectious RNP assembly (Figure 9).

## 5. Conclusions

In closing, this study contributes to the quest to identify new strategies to control HIV-1 replication that complement existing treatments. The ability of RocA to disrupt the assembly of infectious HIV-1 RNPs sharply attenuated replication at an IC_50_ of 10 nM in primary CD4^+^ T cells. RocA is a promising anti-tumor agent [36], suppressing cancer growth at doses comparable to those required to inhibit HIV-1 virion formation with minimal toxicity in mice [37,38,39], suggesting that RocA or related compounds could complement existing anti-HIV-1 therapies. We speculate RocA treatment may be a useful to those infected by HIV-1 in providing an additional means to control this infection. 

## Data Availability

Representative EM images are included in manuscript. More data available upon request.

## Supplementary Materials

The following supporting information can be downloaded at: https://www.mdpi.com/article/10.3390/v16091506/s1, Figure S1: Characterization of the U2OS RevGR FSGagGFP cell line; Figure S2: Reversal of RocA inhibition of HIV-1 virion assembly is dependent on new protein and RNA synthesis; Figure S3: Effect of RNA substrate on eIF4A and CA-NC complexes formed; Figure S4: Additional representative electron microscope images of CA-NC assemblies under different conditions.
