# Supplementary material for: Putting a Kink in HIV-1 Particle Infectivity: Rocaglamide Inhibits HIV-1 Replication by Altering Gag-Genomic RNA Interaction
Source: Viruses. 2024 Sep 23;16(9):1506. doi: 10.3390/v16091506 (PMC11437399; doi:10.3390/v16091506)
Supplement: Supplementary file 1 [file viruses-16-01506-s001.zip › viruses-3127207-supplementary.pdf]

Supplemental Figures

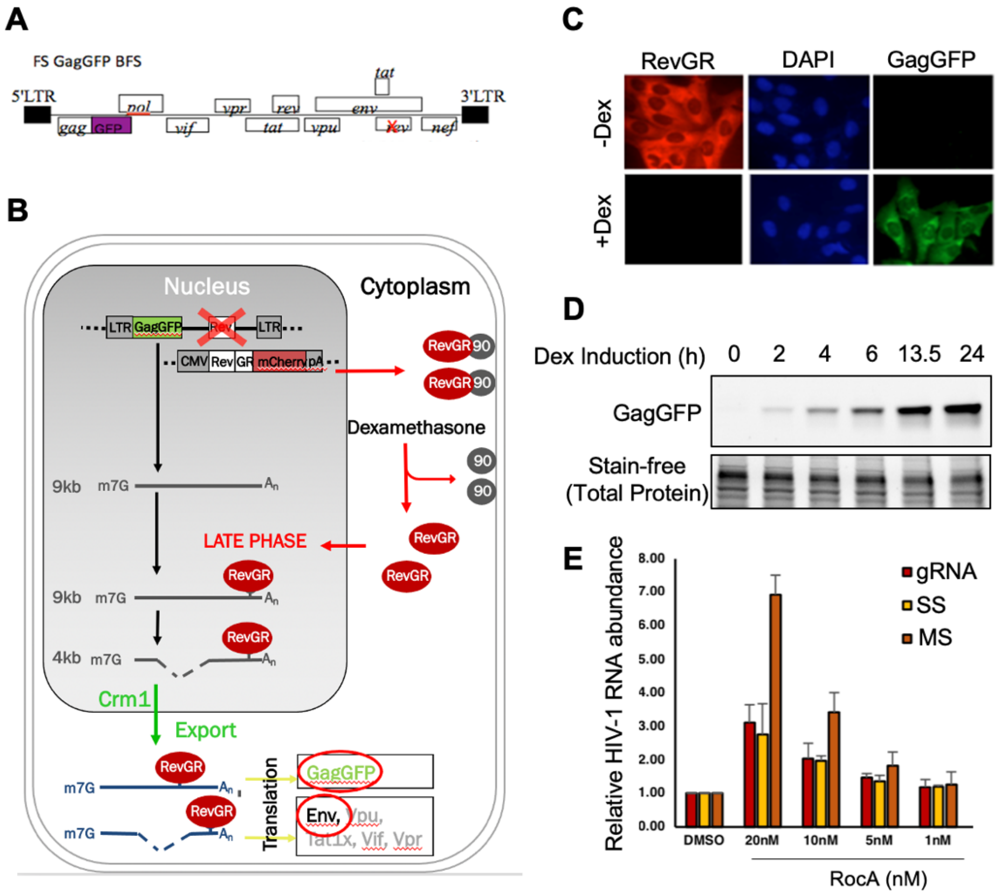

Supplemental Figure S1. Characterization of the U2OS RevGR FSGagGFP cell line. (A) U2OS cells expressing RevGR were transduced with HIV-1 FSGagGFP BFS provirus in which GFP has been fused in frame with Gag (resulting in production of a GagGFP fusion) and the Rev reading frame had been disrupted, rendering virus GagGFP and Env expression dependent on trans expression of Rev. (B) Diagram outlining the regulation of Gag expression of GagGFP expression in the U2OS RevGR FSGagGFP cell line. In the absence of dexamethasone, RevGR is retained in the cytoplasm in association with Hsp90 and export of HIV-1 US/SS RNAs to the cytoplasm is blocked. Upon dexamethasone addition, RevGR is translocated to the nucleus, inducing the export of viral US and SS RNAs to the cytoplasm and GagGF/Env (late phase of virus gene expression) is produced. (C) Cells were fixed after 24 h incubation in the absence (-Dex) or presence (+Dex) of dexamethasone, fixed, stained with DAPI, then stained to detect RevGR and GagGFP.. Magnification 400x. (D) Kinetics of GagGFP expression upon addition of dexamethasone (25 µM). Cells were harvested at indicated times post dexamethasone addition and total cell extracts fractionated on SDS-PAGE cells. Total protein load was imaged (Stain-free, BioRad) then transferred onto PVDF. Gag levels were detected by western blot using anti-Gag antibodies. (E) Cells were treated with dexamethasone for 24h in the absence (DMSO) or presence on increasing doses of RocA. Total RNA was extracted and abundance of HIV-1 US, SS and MS RNAs determined by RT-qPCR as detailed in “Materials and Methods”.

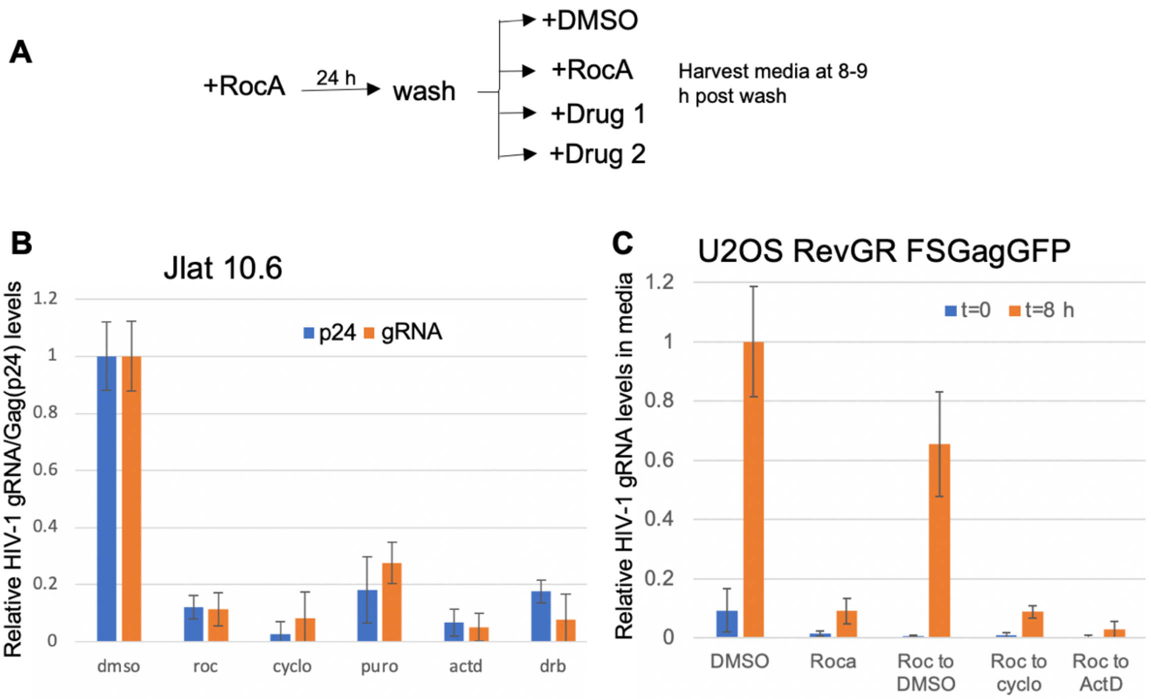

Supplemental Figure S2. Reversal of RocA inhibition of HIV-1 virion assembly is dependent on new protein and RNA synthesis.

(A) Outline of experimental treatment. Cells were incubated with dexamethasone in the presence of 20nM Roca for 24 h. After 24h, cells were washed twice with 1xPBS then media containing DMSO, RocA, or various inhibitors.

(B) In the case of JLat 10.6 cells, new media containing DMSO, RocA, cycloheximide (100 µg/ml), puromycin (2 µg/ml), actinomycin D (actD, 4 µg/ml), or 5,6-dichlorobenzimidazole (DRB) (150 µM) was added. After 8h, cell media was harvested and levels of Gag (p24) and HIV-1 gRNA in media determined by p24 ELISA and RTqPCR, respectively. Results shown are the average of n=3 independent experiments.

(C) U2OS RevGR FSGagGFP cells were incubated with media containing DMSO or RocA for 24 h. Cells were washed and media replaced with fresh media containing DMSO, RocA, cycloheximide (100 µg/ml), or actinomycin D (actD, 4 µg/ml). Aliquots of media were harvested immediately (t=0) or 8h (t=8 h) after media exchange. Levels of HIV-1 gRNA in media were determined by RTqPCR. Results shown are the average of n=3 independent experiments.

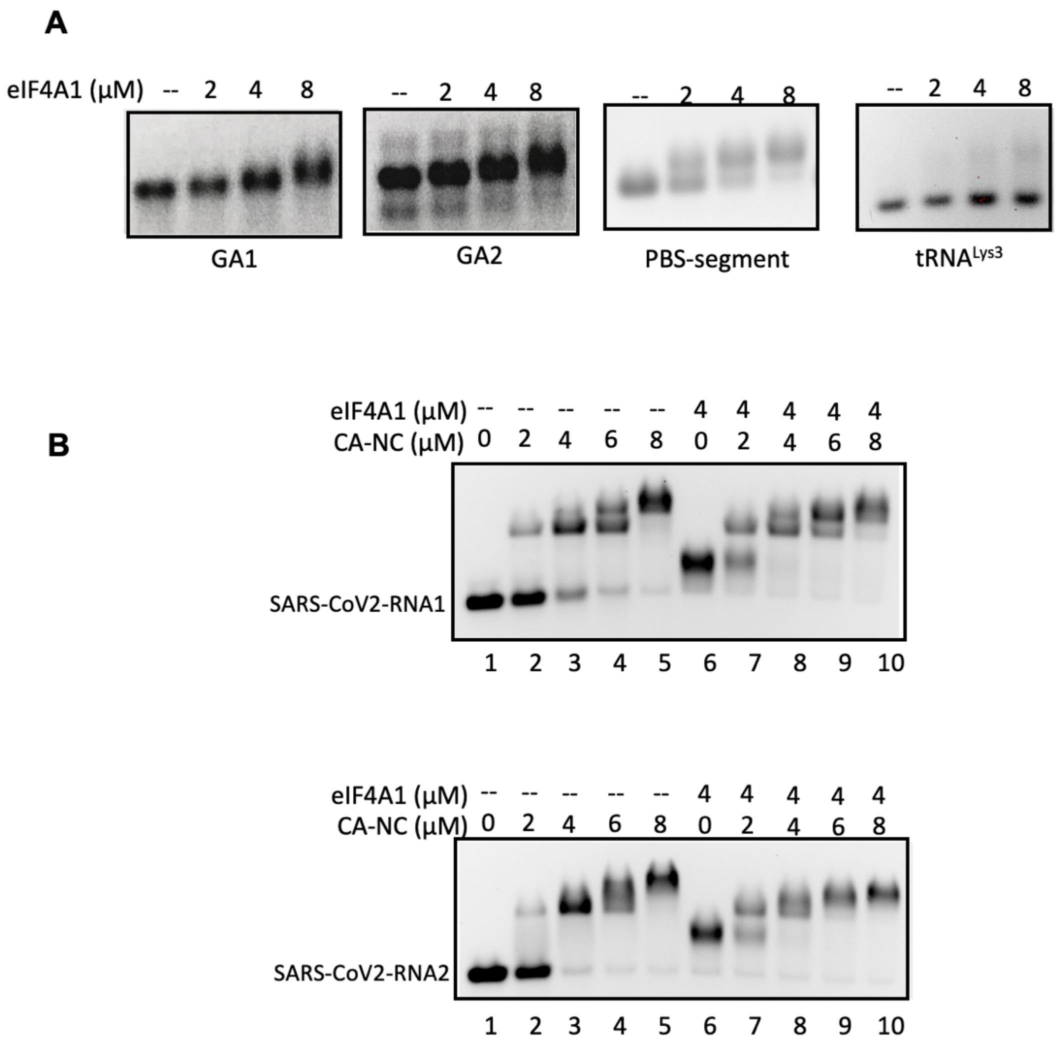

Supplemental Figure S3. Effect of RNA substrate on eIF4A and CA-NC complexes formed. (A) 0-8 µM eIF4A were mixed with GA1, GA2 [22], PBS-segment (nt 125-223), and $tRNA^{Lys3}$ in the presence of RocA and AMP-PNP. The final concentration of each RNA was 0.5 µM. The complexes were resolved on a 1% native agarose gel. (B). CA-NC can bind to non-HIV RNAs pre-bound with eFI4A. Two SARS-CoV2 RNAs at the 3′-UTR were synthesized and purified. 0-8 µM of CA-NC were titrated to these RNA (0.5 µM) in the absence of eIF4A (lanes 1-5), and presence of 4 µM eIF4A (lanes 6-10).

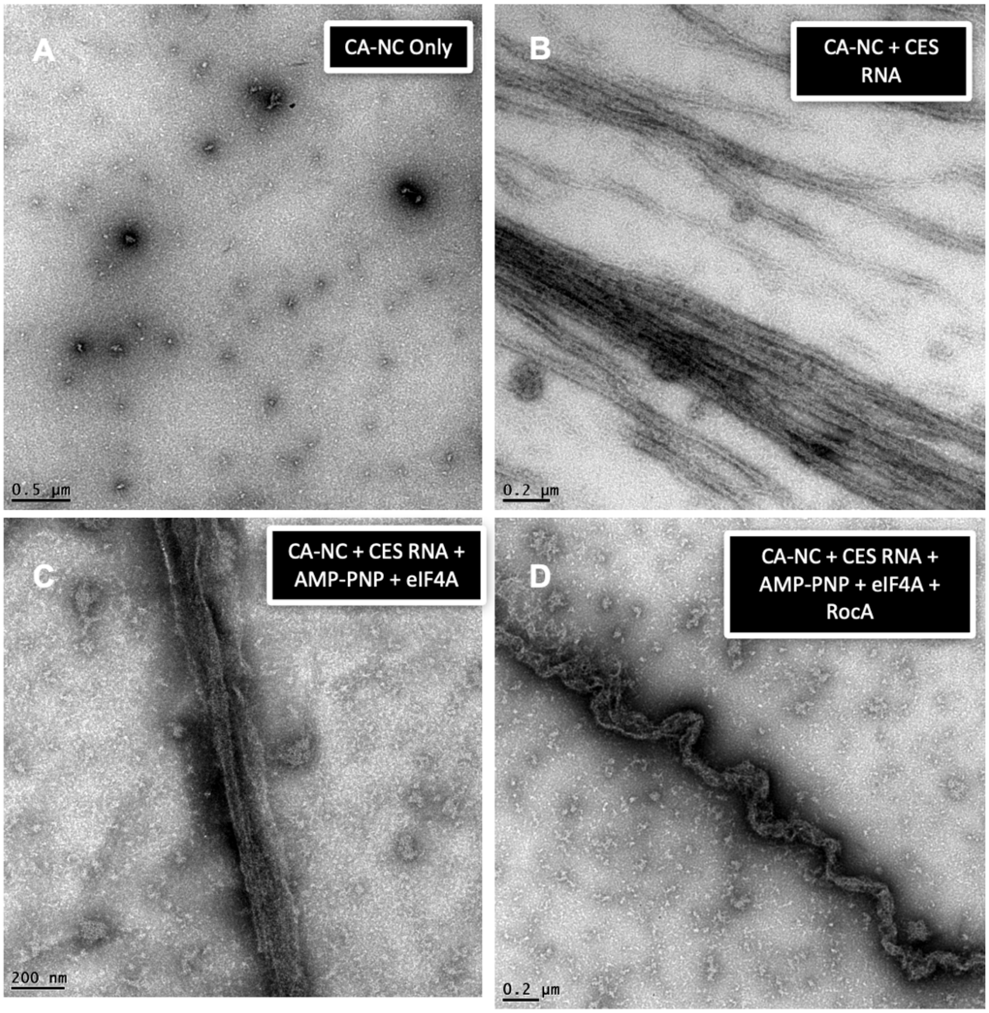

Supplemental Figure S4. Additional representative electron microscope images of CA-NC assemblies under different conditions. A. In the absence of RNA, CA-NC did not form tube structures. B. CA-NC assembled into a lattice structure in the presence of CES RNA. C. CA-NC formed straight tubes in the presence of CES RNA, eIF4A and AMP-PNP. D. The addition of RocA facilitates binding of eFI4A binding to RNA, which introduced kinks to the assembled tube structures.
